# Hybrid Mechanical Vapor Compression and Membrane Distillation System: Concept and Analysis

**DOI:** 10.3390/membranes15030069

**Published:** 2025-02-28

**Authors:** Emad Ali, Jamel Orfi, Salim Mokraoui

**Affiliations:** 1 Chemical Engineering Department, King Saud University, P.O. Box 800, Riyadh 11421, Saudi Arabia; smokraoui@ksu.edu.sa; 2 Mechanical Engineering Department, King Saud University, P.O. Box 800, Riyadh 11421, Saudi Arabia; orfij@ksu.edu.sa

**Keywords:** water desalination, mechanical vapor compression, membrane distillation, hybrid, electrical energy consumption

## Abstract

The concept of integrating mechanical vapor compression (MVC) with direct contact membrane distillation (DCMD) is presented and analyzed. The hybrid system utilizes the DCMD to harvest the thermal energy of the MVC reject brine to preheat a portion of the seawater intake and simultaneously produce additional fresh water. Based on the operating temperature, the hybrid system requires specific energy consumption between 9.6 to 24.3 kWh/m^3^, which is equivalent to 25 to 37% less than the standalone MVC. Similarly, the freshwater production of the hybrid system can range between 1.03 and 1.1 kg/h, which is equivalent to a 3% and 10% increase relative to the standalone MVC when operating at brine temperatures of 50 and 90 °C, respectively. However, this enhancement is achieved at the expense of an average of 60% larger total surface area. This is partially due to the incorporation of the surface area of the MD modules and mostly to reduced temperature differences. Altering the permeate-to-feed ratio of the DCMD module led to a marginal change in the overall production without any enhancement in the compression power consumption. Increasing the MD module length by 50% resulted in a 3% enlargement in the overall production rate and a 10% reduction in power consumption. A modified hybrid structure that additionally utilizes the distillate heat is sought. A 5% increase in water production at the expense of a 45% rise in the specific compression energy of the modified structure over the original hybrid system is obtained.

## 1. Introduction

A shortage of potable water is undoubtedly the most challenging issue globally, as only 30% of the available freshwater is easily accessible. This issue is soaring rapidly due to increasing population and industrialization. It is anticipated to influence about 6 billion people by 2050 [1,2]. Currently, saline water desalination is the most widely used technology to provide drinkable water, especially in Gulf countries such as Saudi Arabia. Desalination technologies can be categorized as conventional and non-conventional or emerging technologies. These methods vary in terms of their worldwide capacity and share as well as their unit desalinated water cost [1,3,4]. Conventional desalination techniques include phase change thermal methods such as multiple-effect distillation (MED), multi-stage flash distillation (MSF), and vapor compression (VC) as well as membrane-based processes such as reverse osmosis (RO) and electrodialysis (ED). These conventional desalination technologies are considered mature, reliable, and can be deployed for large-scale applications where hundreds of thousands of cubic meters of fresh water can be produced daily in a single plant [3,4]. Forward osmosis (FO), membrane distillation (MD), and capacitive deionization (CDI) are examples of emerging desalination processes [1,3]. These processes are still in lab scale or early technological development phases. They need further efforts to solve the main technical problems encountered and find appropriate solutions to their specific limitations. Membrane distillation (MD) stands as a joint thermal and membrane desalination method. MD can be used in a wide range of applications, including sea water and brackish water desalination [3,5], brine concentration [6], wastewater treatment [7], and date juice concentration at low temperature [8]. Boubakri et al. [5] reported that MD can be used to reduce the wastewater flow rejected from various sources, including municipal, textile, pharmaceutical, and oily wastewaters. VMD was also used to produce bioethanol [3].

Energy consumption is a key parameter in considering or selecting a desalination process among others. For example, MSF needs about 10 to 16 (kWh/m^3^), while MED uses about 5.5 to 9 (kWh/m^3^). On the other side, RO energy requirements remain 3 to 4 (kWh/m^3^) for seawater and 0.5 to 2.5 (kWh/m^3^) for brackish water [1]. Similarly, mechanical vapor compression (MVC) needs 7~15 (kWh/m^3^), FO requires 10~68 (kWh/m^3^), ED, which is used most for desalinating brackish waters, demands 1~3.5 (kWh/m^3^), and MD consumes an equivalent of 3~22 (kWh/m^3^) [9]. Criscuoli et al. [4] proposed a clear picture of the energy requirements of various different lab-made flat MD module designs with a 40 cm^2^ membrane area. These numbers of specific energy consumptions (SEC) of various desalination technologies are given just as indicative of the requirements of each process since much higher values have been registered in several operating plants and prototypes. For instance, SEC values higher than 200 kWh/m^3^ have been obtained for several MD lab-scale systems as reported by Najib et al. [10] A comprehensive comparison between these desalination technologies in terms of energy demand, cost of water, advantages, and disadvantages can be found in [11,12,13]. Despite the variation in the extent of energy consumption of these desalination methods, their energy demand still poses a challenge to expansion because of the soaring cost of energy and the stringent environmental restrictions on fossil fuels. This situation forced governments and decision-makers to urge industries to develop sustainable desalination processes. For this reason, investigators proposed the use of renewable energy, mainly solar and wind energy sources, to power desalination plants. Reviews and critiques of using renewable energy for water desalination can be found in review articles [14,15,16]. Other researchers proposed integrating desalination technologies to reduce the existing limitations and capitalize on the merits of the individual systems. Benefits of hybridization in desalination can be found in Nafey et al. [17], Ali et al. [18], Curto et al. [19], and Si et al. [20]. Major merits include a notable increase in the overall capacity and recovery ratio as well as the energy efficiency, while the complexity of the combined structures can result in some operational difficulties [18]. Nafey et al. [17] studied the feasibility of integrating MSF with MED. They concluded that hybridization could reduce the water cost and improve the overall economics compared to the standalone processes. Farsi and Dincer [21] proposed the integration of MED with MD utilizing geothermal energy. They pointed out that most of the exergy destruction takes place in the membrane sheet and the down condenser of the MED. Manesh et al. [22] proposed optimal integration of the existing steam network with a hybrid MED and RO system. They focused on optimizing the steam consumption to power the desalination plant without exploring the structure and design of the hybrid system. Son et al. [23] also focused on the energy utilization in a hybrid desalination plant of MED and the adsorption cycle process. They found that distillate production can be improved by 3~5 folds while using the same energy input.

Very few studies focused on integrating MVC with MD. MVC can be considered a hybrid thermal and electrical process since it is based on the evaporation and condensation of water vapor, but electrical energy is used to drive its compressor and ensure the separation process. MVC has several advantages, such as robustness, compactness, easy integration with classical desalination processes such as MSF and MED, and/or renewable energy sources [24]. Besides, MVC is recognized as one of the few processes that can treat high-salinity solutions [25].

MVC is used in various applications in standalone or coupled with other processes modes. This includes, in addition to the desalination industry, brine management [20] and brine concentration [26]. Randon et al. [27] proposed a techno-economic investigation of a single-effect MVC system in order to minimize the rejected brine volume. Their aim was also to evaluate the thermodynamic behavior and economic viability of the system as a brine concentrator. Despite the important merits of using MVC, its development is facing several limitations. Its high specific energy consumption (SEC) is a major one. SEC values between 23 and 42 kWh/m^3^ have been reported for a single MVC unit [28].

The integration of MVC with MSF has been studied by Mabrouk et al. [29]. They found that the performance ratio of the hybrid structure is 2.4 times that of the single structure of MSF, while the total heat transfer area has increased by 57%. Besides, they concluded that the exergetic efficiency of the hybrid system is 67% higher than the standalone MSF process. Lopez et al. [30] investigated the integration of MED with MVC for zero brine discharge. The hybrid system is powered by wind energy to reduce the cost of compression power. They found that the water cost can be reduced to 0.59 €/m^3^. Zetian et al. [20] investigated the performance of combined vacuum membrane distillation with MVC. The proposed integration achieved better energy efficiency and outstanding economic advantages. Makanjoula et al. [31] studied the integration of a thermoelectric cooler with MD. They showed that leveraging the heating and cooling power of the thermoelectric system can enhance the energy efficiency of the MD. Recently, more efforts towards integrating various types of desalination systems were reported. Bibi et al. [32] studied the integration of vacuum membrane distillation with multi-effect desalination to improve energy efficiency. They concluded that the specific electrical energy can be reduced to 38.42 kWh/m^3^ and that the gain output ratio and production can be enhanced with flow rate and vacuum pressure. Wu et al. [33] investigated the optimization of multi-effect desalination and RO hybrid systems. They found that the hybrid system can reduce energy consumption to 1.015 kgce/m^3^ and increase the recovery ratio by 54.22% at a water cost of 0.56 $/m^3^. Rostami et al. [34] discussed the coupling of humidification-dehumidification, MED, and MVC systems powered by wind energy. They noted that the distillate production improved by 18% and the specific work consumption descends to 21.27 kWh/m^3^ with the aid of the wind energy system. Sawminathan et al. [35] examined the coupling of air gap membrane distillation with MVC, where the MD replaces the brine preheater to recover the heat energy from the hot brine. They pointed out that 6% savings in the water cost can be attained with the benefit of hybridization. However, their investigation focused mostly on the economic analysis.

On the other hand, MD is a promising desalination technology that has several appealing features. For example, MD operates at very low operating temperature and pressure, achieves almost 100% rejection factor, and can be powered by low-grade energy sources [36,37]. Moreover, it can treat a very highly concentrated water solution [38]. However, MD is known for substantial specific energy consumption that can reach 39 kWh/m^3^ [39] of thermal energy.

Schwantes et al. [28] presented a techno-economic analysis of 2 MD variants for zero liquid discharge chain applications. They proposed a comparative cost study with MVC considering the same purpose. Their results show clearly that MD is more cost-effective than MVC, mainly when free waste energy is used to drive the MD process.

With very little effort on MVC-MD hybridization found in the literature, it is of potential to further study the performance and behavior of such integration. By integration, some limitations can be alleviated. For example, the significant energy consumption of MD can be eliminated by harnessing the thermal energy of the MVC distillate and reject brine. In other words, the required energy to drive the MD is provided absolutely for free. In addition, the additional capital cost incurred by using the MD can be offset by the cost of disregarding the heat exchanger responsible for brine preheating. Moreover, the MD performance will not be compromised because it can handle highly concentrated feed. As far as MVC is concerned, the preheaters are used for heat integration, i.e., boosting energy efficiency. Replacing the preheater with MD will maintain the energy efficiency but with the added value of producing additional distillate produced by the MD unit. Another important benefit of integrating MVC and MD concerns the use of two processes identified to treat high-concentration feeds such as brines from conventional desalination technologies, including RO and MED [18,22,34]. Besides, the integration of MD-MVC can contribute to lowering the specific energy consumption, which is still very high for stand-alone MVC systems. Therefore, the frame of this work is to contribute to assessing potential technologies to achieve the minimum liquid discharge (MLD) requirements and move towards more sustainable desalination methods.

The proposed work here is conceptually similar to the work of Sawminathan et al. [35]. However, the objective of this work is to study the design aspects of coupling a single-stage MVC with MD. In particular, the effect of interaction between the two systems will be analyzed. Moreover, the impact of influential design parameters on the overall performance will also be assessed. Specifically, integrating the two systems will create cross-coupling that affects the individual units as well as the overall hybrid system. Investigating the influence of interacting design parameters will reveal the advantages and disadvantages of hybridization.

## 2. Process Description

### 2.1. Mechanical Vapor Compression

**Figure 1:**
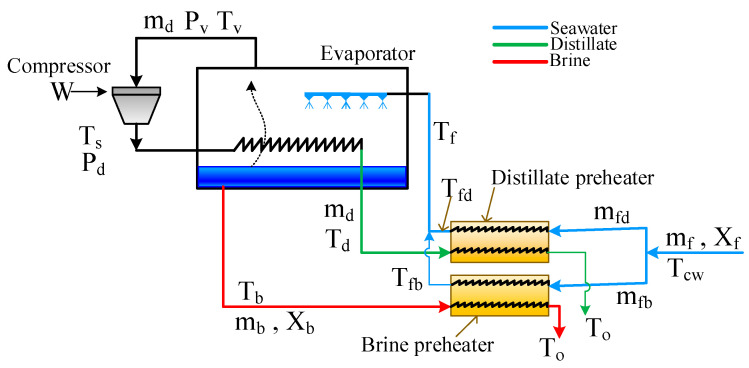
Schematic of mechanical vapor compression.

MVC is a thermal separation process where fresh water is separated from the saline solution by evaporation of the feed water and condensation of the formed vapor by its mechanical compression. The schematic of a typical mechanical vapor compression system is shown in Figure 1. The seawater at temperature T_cw_ and salinity of X_f_ is fed to the preheaters. The distillate preheater uses the thermal energy of the hot distillate to preheat the feed stream. Similarly, the brine preheater uses the thermal energy of the hot brine to preheat the feed stream. As a result, the seawater is heated up to T_f_ and fed to the evaporator chamber. In the evaporator, the feed is sprayed, which quenches the hot compressed vapor inside the condenser coil. When the superheated steam condenses, the brine temperature is raised to T_b_, where a portion of it vaporizes at a rate of *m_d_*. The vapor is sucked at saturation temperature T_v_, which is lower than the brine temperature by an amount equal to the boiling point elevation (BPE), and fed to the compressor. The vapor is then compressed to saturation temperature and pressure of T_d_ and P_d_, respectively. Since the compression is non-ideal, the vapor becomes superheated at temperature T_s_. The superheated steam condenses in the condenser tube as mentioned earlier and cools down to T_o_ in the distillate preheater. The unvaporized brine is pumped into the brine preheater to cool down to T_o_. The conservation equations that describe the process behavior are taken from Ettouney et al. [40] and summarized below. Note that in the work of Ettouney et al. [40], the preheaters are joined in one heat exchanger to facilitate computing the outlet temperature, T_o_, and the feed temperature, T_f_. Here, Figure 1 shows independent heat exchangers to facilitate the incorporation of the MD system when the hybrid system is discussed.

The overall mass and salt balance:

(1) $$m_{f}=m_{b}+m_{d}$$

(2) $$X_{f}m_{f}=X_{b}m_{b}$$

where *m* denotes the mass flow rate in kg/s and *f, b, d* represent the feed, brine, and distillate, respectively. *X* denotes the salinity in ppm.

The heat balance around the preheaters:

(3) $$m_{f}Cp_{f}\left(T_{f}-T_{cw}\right)=m_{d}Cp_{d}\left(T_{d}-T_{o}\right)+m_{b}Cp_{b}(T_{b}-T_{o})$$

In this case, the two preheaters are combined into one, and the outlet temperature for both the distillate and brine streams is assumed to be the same and equal T_o_. Note: *Cp* is the specific heat of the water solution.

The condenser/evaporator heat balance:

(4) $$Q_{e}=m_{f}Cp_{f}\left(T_{b}-T_{f}\right)+m_{d}\lambda_{v}$$

$Q_{e}$ is the amount of heat consumed by the evaporator fluid to raise the feed temperature to the boiling temperature T_b_ and partially vaporize the brine. The latent heat of vaporization is l_v_ at temperature T_v_. The vapor temperature is less than the brine temperature by the boiling point elevation, i.e., $T_{v}=T_{b}-BPE$. This evaporator heat is supplied by the heat of condensation:

(5) $$Q_{c}=m_{d}\lambda_{d}+m_{d}Cp_{v}(T_{s}-T_{d})$$

The condensation energy, Q_c_ is the sum of the sensible heat of cooling the superheated steam from T_s_ to T_d_ and the latent heat of condensation ($\lambda_{d}$) at saturation temperature T_d_. The specific heat of the water solution and the latent heat of vapor are calculated using the correlation in Ettouney et al. [40]. Note that at the condenser, the condensation energy equals the evaporator energy:

(6) $$Q_{e}=Q_{c}$$

As mentioned earlier, the steam leaving the evaporator is compressed from the state (T_v_,P_v_) to a new state (P_d_,T_s_). The compression work is then [24]:

(7) $$W_{a}=\frac{\gamma }{\gamma -1}P_{v}V_{v}\left[{\left(\frac{P_{d}}{P_{v}}\right)}^{\frac{\gamma -1}{\gamma }}-1\right]$$

where g is the specific heat ratio and has a value of 1.42 for water vapor. *V_v_* is the specific volume of the vapor and is calculated at *T_v_* using the correlation in El-dessouky and Ettouney [41]. Similarly, the vapor pressures P_v_ and P_d_ are calculated at the saturation temperatures T_v_ and T_d_, respectively, using the correlation in El-Dessouky and Ettouney [41]. The actual compression power is given as follows:

(8) $$W=\frac{W_{a}}{\eta }=H_{s}-H_{v}$$

where h is the compressor efficiency. *H_v_* is the enthalpy of the saturated vapor at *T_v_*, and *H_s_* is the enthalpy of the superheated steam at *P_d_* and *T_s_*. The specific compression work is given as follows:

(9) $$sW(\frac{kWh}{m^{3}})=\frac{W}{3.6\times m_{d}}$$

The heat transfer area of heat exchangers:

The heat transfer area for the evaporator is given as follows:

(10) $$A_{e}=\frac{Q_{e}}{U_{e}(T_{d}-T_{b})}$$

where U_e_ is the overall heat transfer coefficient for the evaporator and is calculated using the correlation of Ettouney et al. [40]. The heat transfer area for the distillate preheater and brine preheater is computed as follows:

(11) $$A_{b}=\frac{m_{b}Cp_{b}(T_{b}-T_{f})}{U_{b}LMTD_{b}}$$

(12) $$A_{d}=\frac{m_{d}Cp_{d}(T_{d}-T_{f})}{U_{d}LMTD_{d}}$$

where *U_b_* and *U_d_* are the overall heat transfer coefficients for the brine and distillate preheaters, respectively. They are calculated using the correlation in El-dessouky and Ettouney [41]. LMDT is the logarithmic mean temperature difference and is defined as follows for the brine and distillate feed preheaters, respectively:

(13) $$LMDT_{b}=\frac{\left(T_{b}-T_{f}\right)-(T_{o}-T_{cw})}{ln\left(\left(T_{b}-T_{f})/(T_{o}-T_{cw}\right)\right)}$$

(14) $$LMDT_{d}=\frac{\left(T_{d}-T_{f}\right)-(T_{o}-T_{cw})}{ln\left(\left(T_{d}-T_{f})/(T_{o}-T_{cw}\right)\right)}$$

Accordingly, the total heat transfer area and the normalized transfer area are defined as follows:

(15) $$sA_{t}=\frac{A_{t}}{m_{d}}=\frac{A_{e}+A_{b}+A_{d}}{m_{d}}$$

In addition, the following are definitions of key temperature differences that are used for discussion and analysis:

The feed temperature difference:

(16) $$\Delta T_{f}=T_{b}-T_{f}$$

The condenser temperature difference:

(17) $$dT=T_{d}-T_{b}$$

The steam temperature difference:

(18) $$\Delta T_{s}=T_{s}-T_{d}$$

### 2.2. The Hybrid System

**Figure 2:**
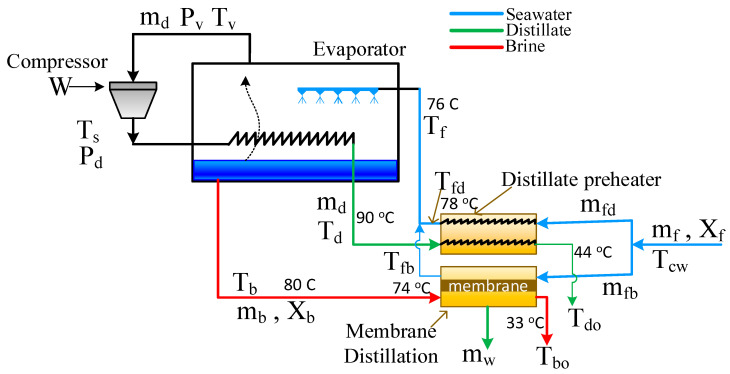
Schematic of the hybrid MVC-MD system (S1).

The Hybrid System is displayed in Figure 2 and denoted as S1. Simply, the brine preheater is replaced by the MD module. Therefore, the MD has dual functions; one is to preheat the seawater feed, and the second is to produce additional fresh water at the rate of *m_w_*. The advantage of this integration is that the MD harnesses the free energy of the reject brine to produce more water and simultaneously heat the feed. It should be noted that regular DCMD uses distillate water as a permeate stream, also known as a cold stream. However, the permeate stream in this structure is the seawater. Hence, the permeate gap membrane distillation (PGMD) is suitable in this case because the water flux is collected and withdrawn separately from the seawater stream. As far as the distillate preheater is concerned, it cannot be replaced by an MD module because, in this case, the hot stream is the distillate stream, which does not need to be further purified. The mathematical equations describing the hybrid system comprise the model equations of MVC and MD. The model of MVC remains the same as mentioned in Section 2.1 above.

The mathematical model of the MD is a combination of coupled mass and energy transport equations as described in Appendix A. The MD module represents the DCMD pilot plant provided by Solar Spring, Germany. The membrane is constructed as a spiral wound made of polyethylene tetrafluoride. The membrane characteristics and full description of the process are given elsewhere [42,43,44].The model was previously developed and validated using experimental data [43,44,45]. The numerical solution of the MD model given in Appendix A requires the feed conditions, i.e., the flow rate, temperature, and salinity of the feed and permeate streams ($m_{b},\;m_{fb},\;T_{b},\;T_{cw},\;X_{b}$). The output of the model is thus the outlet temperatures of the feed and permeate streams ($T_{o},\;T_{fb})$ and the water production, *m*_w_. It should be noted that the model equations are developed for DCMD, but they will be assumed to resemble the same performance of PGMD. For the overall system, the total water production will be as follows:

(19) $${m_{d}}_{t}=m_{d}+m_{w}$$

Hence, the specific compressor work and the specific total area given by Equations (9) and (15) should be scaled by ${m_{d}}_{t}$. Moreover, the total required surface area shall be augmented as follows:

(20) $$A_{t}=A_{e}+A_{d}+A_{MD}$$

Accordingly, the specific total area in Equation (15) should be updated to include the contribution of the MD module as described by Equation (17). Note: when a hybrid system is used, the total area does not include the brine preheater. It should also be noted that a single MD module has 10 m^2^ of effective area, which represents the actual module used to validate the model. Note also that a DCMD model is used to resemble PGMD. Usually, PGMD has a lower production rate for the same surface area [46]. However, for the sake of simplicity, we will overlook this point as the comparison of the standalone MVC with the hybrid MVC is relative. Moreover, in this study, cold seawater will be used in the cold side of the MD instead of permeate. However, the word permeates will be used interchangeably with seawater to be consistent with the common terminology of MD technology.

## 3. Simulation Procedure

In this section, the algorithms to solve the model equation of the standalone MVC and that of the hybrid system will be presented. First, the algorithm adopted by Ettouney et al. [40] to solve the MVC will be explained and denoted as Section 3.1. A modified algorithm to solve the MVC alone and the hybrid system will also be explained. This algorithm will be denoted as Section 3.2.

### 3.1. Algorithm A1

The solution of the MVC model (Equations (1)–(15)) is based on the design specification listed in Table 1. This means the design specification is the production of 1 kg/s of distillate with a reject brine concentration of 70,000 ppm. In addition, *T_b_* and *T_d_* will be prespecified. Hence, *m_b_*, *m_f_*, *T_f_*, *T*_o_, and *T_s_* are left as unknowns. The solution procedure, which is taken from Ettouney et al. [40] is described as follows:

1. Using Equations (1) and (2), find *m_b_* and *m_f_*_._
2. Using Equation (7), find the isentropic compressor work. Using Equation (8) and the preassigned compressor efficiency, find the actual work and *T_s_*.
3. Using Equations (3) and (6), find *T_f_* and *T*_o_ iteratively.
4. Compute the normalized transfer area using Equations (10)–(15).

According to this simulation procedure, the outlet temperatures of the preheaters (*T_f_*, *T_o_*) are adjusted to satisfy the design specification. This means the performance (efficiency) of the heat exchanger is adjustable and variable to meet the required design specifications. In reality, the performance of the heat exchanger cannot be adjusted for fixed inlet temperature and flow rates. In addition, this solution procedure is not suitable for the hybrid system as the outlet temperature of the MD module is controlled by the coupled heat and mass operation inside the membrane. For this reason, another solution algorithm will be used as explained next.

### 3.2. Algorithm A2

In this algorithm, *T_b_* and the efficiency of the preheater heat exchangers will be specified. The rest of the variables will be determined iteratively. In fact, *T_d_* will be adjusted to satisfy the design specifications. The algorithm can be explained by the following procedure:

1. Using Equations (1) and (2) find *m_b_* and *m_f_*_._
2. Assume a value for *T_d_*
3. Given the heat exchanger efficiency solve the following heat balance to find *T_f_* and *T_o_*:
  - For the distillate preheater find:
    - $Q_{h}=\eta_{h}\times min(m_{d}Cp_{d},m_{fd}Cp_{f})\times \left(T_{d}-T_{cw}\right)$
    - $T_{fd}=T_{cw}+\frac{Q_{h}}{m_{fd}Cp_{f}}$
    - $T_{do}=T_{d}-\frac{Q_{h}}{m_{d}Cp_{d}}$
  - For the brine preheater find:
    - $Q_{h}=\eta_{h}\times min(m_{b}Cp_{b},m_{fb}Cp_{f})\times \left(T_{b}-T_{cw}\right)$
    - $T_{fb}=T_{cw}+\frac{Q_{h}}{m_{fb}Cp_{f}}$
    - $T_{bo}=T_{b}-\frac{Q_{h}}{m_{b}Cp_{b}}$
  - Compute the weighted average feed temperature: $T_{f}=m_{fd}T_{fd}+m_{fb}T_{fb}$
4. Using Equation (7), find the isentropic compressor work. Using Equation (8) and the preassigned compressor efficiency, find the actual work and *T_s_*.
5. Check the equality given by Equation (6); if it is satisfied, stop the iteration.
6. Otherwise, update *T_d_* and repeat steps 3 to 5.

This algorithm will be used for solving the stand-alone MVC and the hybrid system. In the case of the hybrid system, step 3b is replaced by solving the MD module equations to obtain *T_fb_*. For both cases (MVC, Hybrid), the partitioning of the seawater stream to feed the preheaters is defined as follows:

(21) $$m_{fb}=m_{b}$$

(22) $${m_{f}}_{d}=m_{d}$$

This is the simplest division of the seawater stream and renders the best performance for each heat exchanger. Moreover, for the hybrid system, the maximum feed flow rate to a single MD is taken as 500 L/h. Although Triki et al. [47] reported a maximum feed flow rate of 1650 L/h, we limited the analysis to 500 L/h to avoid pore-wetting conditions. Therefore, for the given capacity of the given process in this study, three MD units in parallel will be utilized. Accordingly, the total production and the total surface area of the MD system will be threefold of that computed for a single MD unit. The characteristics of the MD membrane to be used in this study are listed in Table 2.

## 4. Results and Discussion

### 4.1. Model Validation

**Figure 3:**
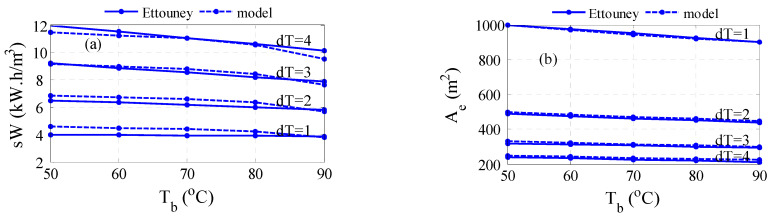
Comparison of the model prediction with published data: (**a**) specific work, (**b**) evaporator area of heat transfer.

**Figure 4:**
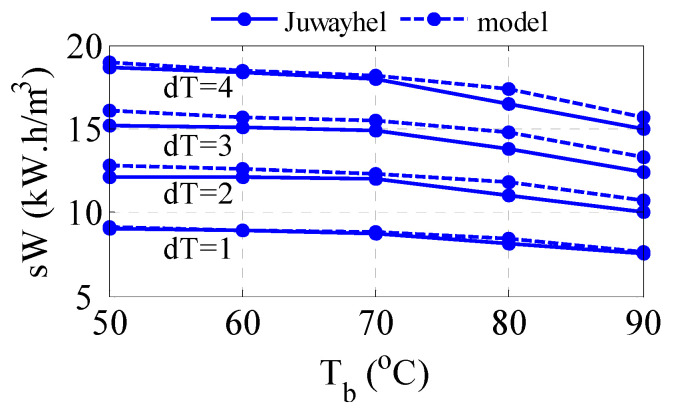
Comparison of the predicted specific work with published data.

First, the model of the MVC is validated against published data. Specifically, the data published by Ettouney et al. [40] is used here. The operating conditions for this process are listed in Table 1. Figure 3 illustrates the comparison result over a range of values for the evaporator temperature, *T_b_*, and selected value for *dT*. Algorithm A1 is used here. Specifically, Figure 3a shows the fitting of the predicted specific work of the compressor with that of the reported data. The accuracy of the model prediction is acceptable with a maximum percent error of 14%. Figure 3b depicts the fitting of the predicted heat transfer area of the evaporator with that of the reported data. Perfect verification of the model accuracy is obtained with a maximum error of 3%. The profile of the specific work decreases with brine temperature for each fixed condensing temperature, *T_d_*. It is known that at higher brine temperatures, the tendency to evaporate is higher; consequently, the required energy for evaporation and thus compression work will decrease. At higher condenser saturation temperatures, i.e., increasing *T_d_*, which is manifested by increasing *dT*, will incur higher compression work, which is intuitive. The profile of the evaporator follows the same trend of work with respect to *T_b_*. As the condenser energy decreases, the required heat transfer area of the evaporator will diminish accordingly. However, since the heat transfer area is inversely proportional to the temperature difference across the condenser tube, i.e., *dT* = *T_d_* − *T_b_*, the resulting area of the evaporator will decrease with *dT*. Apparently, the reported specific work changes linearly with *T_b_* while the predicted one exhibits downward curvature. In fact, the trend of the compression work is expected to behave nonlinearly with temperature as the vapor pressure and specific volume of the vapor change nonlinearly with temperature. Indeed, the reported specific work of Juwayhel et al. [48] demonstrates a monotone reaction with temperature as depicted in Figure 4. In this case, the model prediction shows better verification of the reported data with a maximum error percentage of 7%. The minor mismatch between the model output and reported data can be attributed to the type of correlations used to estimate the physical properties and the accuracy of the numerical technique. It should be noted that the reported data in Figure 3 and Figure 4 are estimated from the given graphs in these references as no numerical data are available. This creates uncertainty and increases the mismatch between the model prediction and the reported data. Nevertheless, the model is considered trustworthy for conducting further analysis.

### 4.2. Preheater Analysis of the Standalone MVC

In this section, the reflection of the simulation procedure (Algorithm A1) on the performance of the feed preheaters is demonstrated. The simulation procedure fits the outlet temperature of the preheaters (*T_f_* and *T_o_*) to meet the designed capacity of the condenser, i.e., preassigned compression work as both *T_b_* and *T_d_* are prespecified. This makes the efficiency of the preheater heat exchanger variable; in other words, the heat exchanger efficiency is regulated to satisfy the required distillate production and its associated compression work.

**Figure 5:**
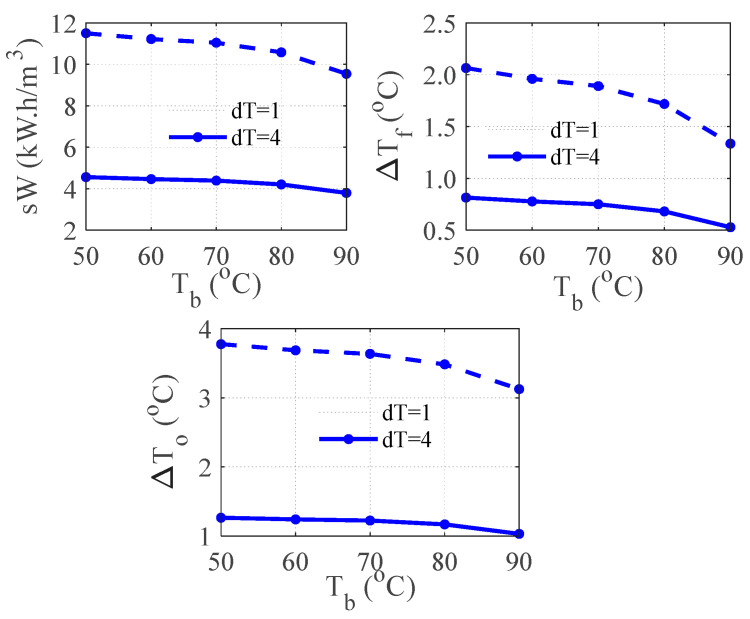
Effect of Temperature difference (dT) on the outlet temperatures for standalone MVC; $\Delta T_{f}=T_{b}\;{-\;T}_{f}$, $\Delta T_{o}=T_{o}-T_{cw}$.

Figure 5 shows the predicted compression work along with the associated outlet temperatures of the heat exchanger. The differenced temperatures are shown, i.e., the difference between the top outlet temperature *T_f_* and the corresponding inlet temperature of the brine, as well as the difference between the bottom outlet temperature *T_o_* from the corresponding inlet temperature of the seawater *T_cw_.* For each data point in the figure, *T_b_* and *T_cw_* are specified while *T_f_* and *T_o_* are computed from the heat balance around the heat exchanger. We can see that the profile of the required work follows the trend of D*T_f_*. As D*T_f_* decreases with *T_b_*, the required energy to heat the feed and evaporate the brine becomes smaller as well. It should also be remembered that the ratio of the vapor pressure at the saturation temperature *T_d_* and *T_b_* declines with *T_b_*. Moreover, the differences D*T_f_* and D*T_o_* become very small, especially at *dT* = 1. In fact, D*T_f_* becomes less than 1. This makes the required transfer area of the preheater soar considerably. In addition, this situation incurs a large and variable efficiency of the heat exchanger. The computed efficiency of the heat exchanger at *dT* = 1 ranges between 94% and 97%, which is unrealistic. For this reason and to integrate MD with MVC, a different simulation procedure (Algorithm A2) will be adopted as mentioned earlier.

### 4.3. Comparison of the Hybrid System with MVC

**Figure 6:**
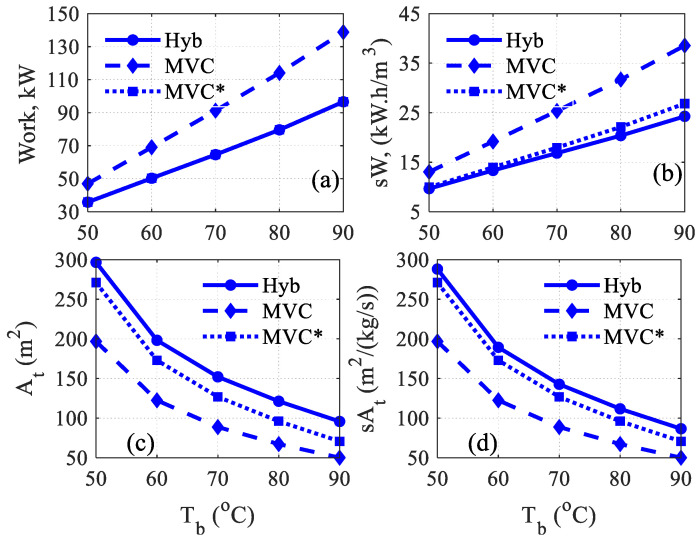
Comparison of the performance of the hybrid system with MVC at fixed heat exchanger efficiency of 80%; MVC* stands for MVC standalone using the heat transfer efficiency of MD; (**a**) Work, (**b**) Specific work, (**c**) Total area, (**d**) Specific total area.

**Figure 7:**
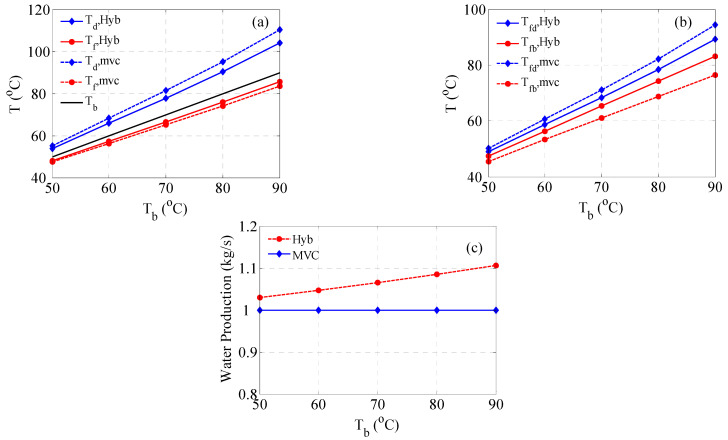
Temperature profile and water production associated with the results in Figure 6; (**a**) Feed and brine temperatures, (**b**) Distillat and brine preheaters temperatures, (**c**) Water production.

In this section, the performance of the hybrid system (MVC + MD) will be compared with the standalone MVC system. The simulation procedure represented by algorithm A2 will be utilized for both systems for fair comparison. In this case, the efficiency of the preheater heat exchangers will be fixed at 80%. The rest of the process parameters will remain as listed in Table 1. Figure 6 and Figure 7 display the comparison result over a range value for the brine temperature. Note that *dT* ($dT=T_{d}-T_{b}$) is not preassigned because the saturation temperature of the condenser ($T_{d}$) is determined via an iterative procedure. For given *T_b_* and *m_d_*, the vapor saturation temperature is determined such that the evaporator energy equals the condenser energy. Recall the required separation is manifested by *m_d_* = 1 kg/s, and the brine salinity is 70,000 ppm. Figure 6a displays the required compression energy, and the associated specific energy is shown in Figure 6b. The hybrid system demands less energy for the same operating conditions. In fact, the hybrid system requires 32.5% less average compression power. The reason for this improvement is related to the effect of MD on D*T_f_*, as will be shown and discussed in Figure 7a.

The normalized work of MVC shown in Figure 6b remains equal to the raw work because the production is 1 kg/s; however, the magnitude is scaled down due to unit conversion. Besides unit scaling, the normalized work of the hybrid system is slightly reduced because of the increasing overall production as shown in Figure 7c. The effect of MD production on the specific work is minute because the increase in the overall production is also minor. As depicted in Figure 6c, the total required heat transfer area is larger for the hybrid system. The average increment in the area over the range of operating *T_b_* is around 60%. The reason for the inflated transfer area of the hybrid system is the smaller temperature difference, *dT*, as shown in Figure 7a. Moreover, the total area of the hybrid system includes the surface area of the MD, which is constant at 30 m^2^ since three modules are used. Figure 6d depicts the normalized heat transfer area. Of course, the specific area of MVC remains the same, while that for the hybrid system is slightly scaled down because of the increased overall production. Nevertheless, the specific area of the hybrid system is still larger than that of MVC because of the minor contribution to the overall production.

Figure 7a demonstrates the temperature distribution for the feed and condenser for both systems. Note that *T_d_* is not preassigned here but variable and that *T_f_* depends on *T_b_*, *T_d_*, and the heat exchanger efficiency. For MVC, D*T_f_* is wider, incurring larger *T_d_* and subsequently larger compression energy. Conversely, for the hybrid system, D*T_f_* is narrower, necessitating less compression work. This is the direct conclusion of Equations (4) and (5). When D*T_f_* is large, higher evaporator energy is needed, which is also reflected in the condenser energy. Since the latent heat at *T_d_* is always smaller than that at *T_v_*, larger temperature difference D*T_s_* are necessary. This explains why the compression power for MVC is higher than that of the hybrid system, as was shown in Figure 6a. The difference between D*T_f_* for MVC and that for the hybrid system is further explained in Figure 7b. Indeed, Figure 7b shows the profile of the feed temperature exiting the distillate preheater (*T_fd_*) and the feed temperature exiting the brine preheater (*T_fb_*). *T_fd_* of MVC is larger than that of the hybrid system, although the preheater works at the same efficiency for both systems. The reason is that the corresponding *T_d_* for MVC is higher than that of the hybrid system, as was clearly shown in Figure 7a. On the other hand, *T_fb_* of the hybrid system is much higher than that of MVC because it is the result of the MD performance. In a typical MD system, the permeate stream exits the MD within 2~3 degrees of the feed inlet temperature, which is *T_b_* in this case. Thereby, *T_f_*, which is the weighted average of *T_fd_* and *T_fb,_* is higher for the hybrid system. Therefore, to reduce the total surface area requirement in the hybrid case, $\Delta T_{f}$ should be widened, which can be achieved by raising $T_{f}$. The latter can be enlarged by increasing the efficiency of the distillate preheater to boost $T_{fd}$ and/or increasing the heat transfer efficiency of the MD to boost $T_{fb}$. The latter can be attained either by elevating the circulation rate or using a longer membrane, which is not recommended because it will also increase the surface area. Nevertheless, attempts to reduce the surface area will enjoin escalation in the compression work. Hence, there is a trade-off between energy efficiency and surface area.

Recall that, in this study, the ratio of the mass rate of the brine to the distillate rate is 1.5:1, which gives more weight to *T_fb_*. This situation may differ if the heat exchanger of the brine preheater has a higher efficiency, as will be discussed later. Finally, Figure 7c shows the water production for both systems. In this case, for MVC, the water production is fixed at 1 kg/s by design. However, the water production of MD varies with feed temperature, i.e., *T_b_*, which augments the overall production. The growth of water production of MD with feed temperature is well known as it raises the driving force and consequently the mass flux [37,49,50]. However, the increment in the overall production is minor, which corresponds to 5% at the lowest temperature and 10% at the highest operating temperature. The minor water production of MD is also common as the recovery ratio is between 5 and 10% for a single pass [51,52,53,54]. This explains the minor impact of the MD production on the normalized work and normalized area that was shown in Figure 6.

The discrepancy between the performance of the hybrid system and the standalone MVC shown in Figure 6 is related to the difference in the feed temperature ($T_{f}$) as shown in Figure 7a. This behavior is attributed to the disparate heat transfer effectiveness between the MD and preheater. The MD has higher heat transfer efficiency, leading to greater feed temperature, which entails better overall energy efficiency manifested in lower compression work. For fair comparison, the standalone MVC is operated using the same feed temperature generated by the MD unit. The results are depicted in Figure 6 using a dotted line and denoted MVC*. In this case, the MVC ordains the same amount of compression work required by the hybrid system. However, the specific work is slightly higher because the hybrid system has an elevated production rate, especially at high operating temperatures. Hence, the use of MD provides an effective heat exchange mechanism and leads to additional distillate production. Alternatively, a brine preheater with higher efficiency is needed to improve the MVCs energy efficiency. However, increasing the preheater effectiveness will be at the cost of a higher transfer area, as illustrated in Figure 6c,d. This confirms that any attempts to enhance the energy efficiency will incur higher capital costs.

The above analysis highlighted the importance of considering hybrid desalination systems. The presented results are aligned with the general trend that hybrid structures lead to a substantial decrease in the specific energy consumption, but the total heat (mass) transfer area could be increased.

### 4.4. Effect of the Heat Exchanger Efficiency

**Figure 8:**
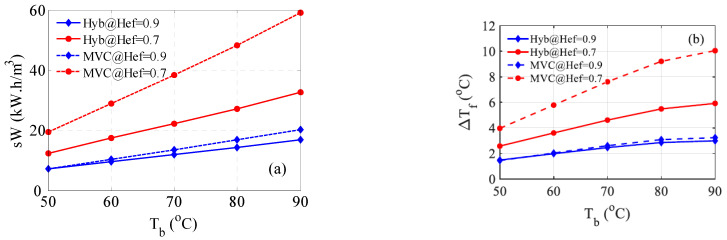
Effect of the heat exchanger efficiency (Hef) on the performance of MVC and the hybrid system; (**a**) Specific work, (**b**) $\Delta T_{f}=T_{b}-T_{f}$.

As discussed earlier, the efficiency of the heat exchanger has a direct influence on the feed temperature and hence on the performance of both MVC and the hybrid system. Therefore, the effect of varying the heat exchanger efficiency is studied. The results are shown in Figure 8 for 70% and 90% efficiency. At lower efficiency (70%), both systems’ performance in terms of compression power required deteriorates, i.e., becomes larger. Nevertheless, the hybrid system still outperforms MVC. This is because the hybrid system is partially influenced by the heat exchanger’s efficiency via the distillate preheater, while the other part (*T_bf_*) is controlled by the MD performance. Bearing in mind that *T_bf_* has more weight, D*T_f_* becomes smaller as shown in Figure 8b. On the other hand, at a high efficiency of 90%, both systems’ performance improves, and the hybrid system still dominates for the same reason discussed above. Note that the compression work of the hybrid system at 90% efficiency is enhanced, i.e., reduced by 28% on average compared to that of the hybrid system at 80% efficiency. Similarly, the work of the MVC system at 90% efficiency is enhanced, i.e., reduced by 20% on average compared to that of the hybrid system at 80% efficiency. Figure 8b illustrates the feed temperature difference at 70% and 90% efficiency. It is evident that D*T_f_* is always smaller for the hybrid system for the reason discussed earlier. Moreover, the difference in D*T_f_* between MVC and the hybrid system is modest at 90% efficiency but has a greater reflection on the compression work. This is attributed mainly to the exponential variation of the vapor pressure with temperature, as the augmented production rate has a minimal contribution. In addition, for 90% efficiency, both systems have comparable energy of compression at low brine temperatures of 50 and 60 °C. This can be ascribed to the reduced efficiency of the MD at low temperatures.

### 4.5. Effect of the Flow Rate Ratio

Usually, the ratio of the cold stream flow rate to the hot stream flow rate affects the MD performance in two ways. First, it influences the permeate outlet temperature because the thermal capacitance of the two streams becomes unequal when the ratio is not equal to one. Secondly, it affects the distillate mass flux. Some researchers found that the maximum water flux occurs at equal flow rates [55,56]. While Line et al. [57] reported an optimal flux occurs at a 90~92% ratio. Naidu et al. [58] also found that a permeate to feed ratio of 1.4 maximizes the flux. Ali [59] also reported a maximum flux that may occur at a flow rate ratio of 0.7. Therefore, it is interesting to study the effect of the ratio of the permeate to the feed flow rate on the performance of the hybrid system. Note that altering the flow rate ratio will also affect the portion of the feed (seawater) flow rate forwarded to the distillate preheater. Hence, the flow rate ratio is expected to impose a combinatorial impact on the overall process. The flow rate ratio is defined as follows:

(23) $$R=\frac{m_{fb}}{m_{b}}$$

The nominal case for *R* is 1, i.e., $m_{fb}=m_{b}\;$ as described previously in Section 3.2.

**Figure 9:**
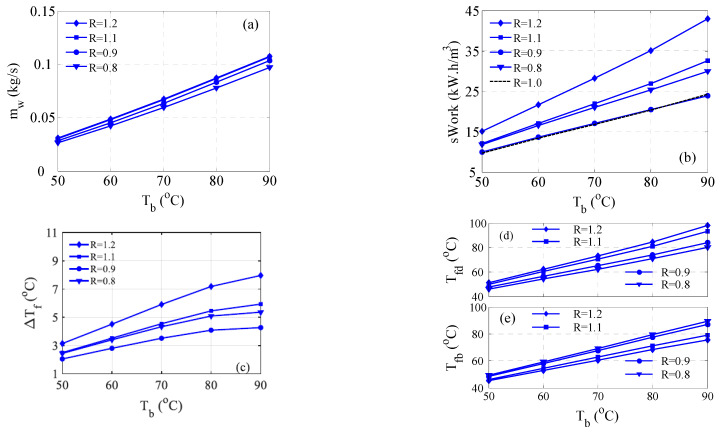
Effect of the ratio of permeate to feed flow rate on the performance of the hybrid system; (**a**) Water production, (**b**) Specific work, (**c**) $\Delta T_{f}$, (**d**) Distillat preheater temperature, (**e**) Brine preheater temperature.

Figure 9 depicts the performance of the hybrid system for a flow rate ratio between 0.8 and 1.2. Figure 9a displays how the water production of the MD unit varies with the ratio. Generally, the water production increases with *R* but becomes almost equal at ratios of 1.1 and 1.2. Moreover, the variation of *m_w_* with *R* is minor. Note that the effect of the flow rate ratio on the MD performance is limited by the feed salinity extent, the inlet cold temperature, and membrane characteristics. Figure 9b depicts the variation of the specific compression power with *R*. Notably, the power rises with *R*, but the lowest occurs at *R* = 0.9. Unfortunately, the minimum work energy coincides with that obtained at equal flow rates. Hence, altering the flow rate ratio is not beneficial. As discussed earlier, the required work is heavily related to D*T_f_*, which is shown in Figure 9c. Apparently, the feed temperature difference grows with *R*, but the minimum occurs at *R* = 0.9. The reason for that is explained in Figure 9d,e. As demonstrated by Figure 9d, as the ratio increases, a smaller portion of the seawater is forwarded to the distillate preheater, which makes the outlet seawater temperature (*T_fd_*) soar. Note that the distillate temperature is even higher in these cases. On the other hand, as *R* increases, the outlet seawater temperature (*T_fb_*) shown in Figure 9e decreases because the thermal capacitance of the permeate stream enlarges and its residence time lessens. For this reason, the smallest weighted average of *T_fb_* and *T_fd_* takes place at *R* = 0.9.

### 4.6. Effect of the Membrane Length

**Figure 10:**
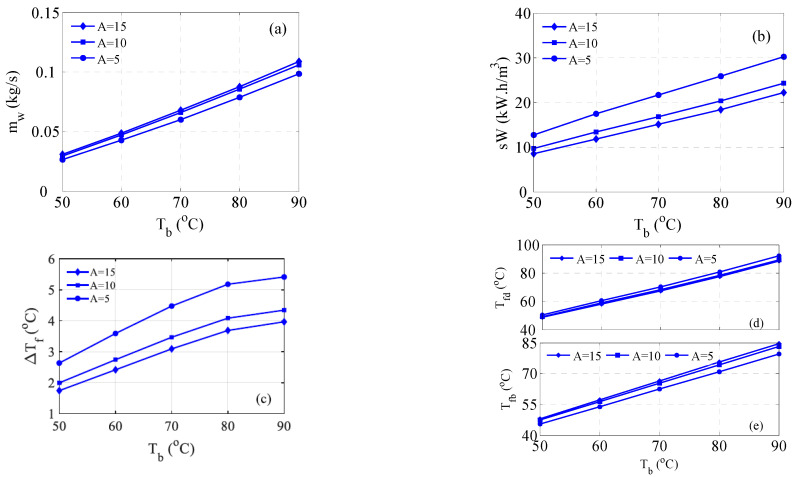
Effect of MD Module surface area on the performance of the hybrid system; (**a**) Water production, (**b**) Specific work, (**c**) $\Delta T_{f}$, (**d**) Distillate preheater temperature, (**e**) Brine preheater temperature.

The MD membrane length manifested by its corresponding surface area influences the MD performance. Note that the surface area is proportional to the module length. A Longer MD module, i.e., higher surface area, reduces the mass flux but enlarges the overall production. Besides, a longer module increases the contact time, and consequently, the permeate departs the module at a slightly higher temperature. Hence, it is of potential to investigate the effect of the membrane length on the hybrid system performance. Figure 10a shows how *m_w_* evolves with surface area, with the highest belonging to A = 15 m^2^. Nevertheless, the evolution of the production with area remains marginal. The average increment in *m_w_* at the highest area is 3%. Concerning the specific power required, it becomes smaller at longer modules as shown in Figure 10b. The average enhancement in the specific work is around 10% at the highest surface area. This is attributed to the slightly enhanced production (Figure 10a) and reduced feed temperature difference as shown in Figure 10c. As shown in Figure 10c, the smallest D*T_f_* occurs at the largest surface area. This is due to the feed temperature profiles displayed in Figure 10d,e. For the short module, the permeate exits the module at a lower temperature as shown in Figure 10e, and vice versa. Since T_fd_ is marginally affected by the surface area, the maximum feed temperature and consequently the smallest D*T_f_* take place at A = 15 m^2^*_._* The reason for the variation of *T_fd_* with the surface area and that it is smaller for larger *A* is explained as follows. As D*T_f_* becomes smaller, the corresponding required distillate temperature (T_d_) lessens as well. As a result, *T_fd_* diminishes accordingly_._ Recall that the simulation of the hybrid system equations is solved iteratively.

### 4.7. Performance of Modified Hybrid Structure

**Figure 11:**
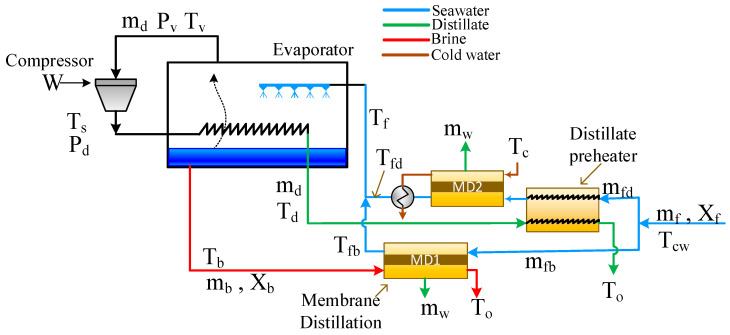
Modified structure for the hybrid system (S2).

In the previous hybrid structure (S1), only the energy of the reject brine is harnessed. It might be interesting to leverage the energy of the distillate stream as it comes out at a higher temperature than that of the brine. However, utilizing the distillate stream directly in the MD process is useless because it is already pure water. Therefore, its energy can be used indirectly to power additional MD units as shown in Figure 11. The modified structure in Figure 11 is denoted as S2. In this case, the warm seawater leaving the distillate preheater is fed to a second MD unit. Auxiliary cold water at temperature *T_c_* is used as the condenser stream. T_c_ is taken to be 25 °C in this study. The seawater passing through MD2 will produce additional distillate (m_w2_) and exit at lower temperatures. The exiting seawater stream is then heated by recovering the heat associated with the outlet condenser stream. This step is essential to ensure that the combined stream entering the MVC is fed at a reasonably high temperature.

**Figure 12:**
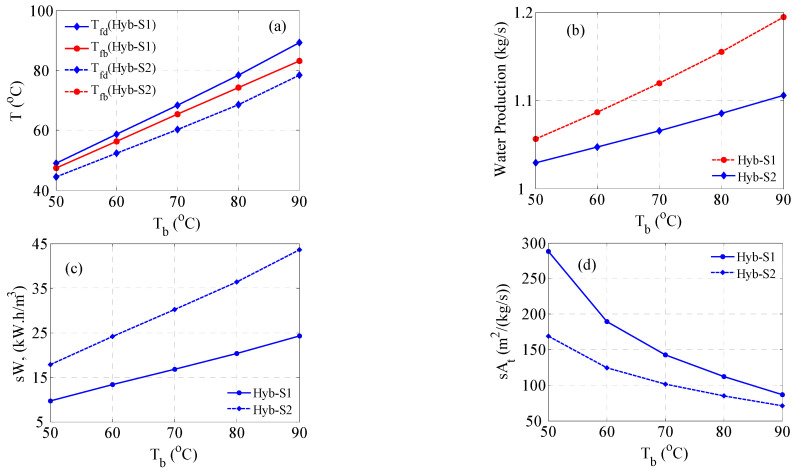
Comparison of Hybrid Structure S1 with S2; (**a**) Temperature, (**b**) Water production, (**c**) Specific work, (**d**) Specific total area.

The results of simulating structure S2 are displayed in Figure 12. Specifically, Figure 12a shows the temperature profile of *T_fb_* and *T_fd_* for both structures. The profile for *T_fb_* remains exactly the same for both structures because this part is not modified. However, the profile of *T_fd_* differs because of modification. In structure S1, *T_fd_* is the outcome of the distillate preheater, while it is the outcome of the recovery system of MD2 in structure S2. Definitely, *the T_fd_* for S2 is lower than that for S1. Note the seawater entering MD2 will lose a large amount of energy to produce the distillate. Although it is later heated in the recovery system, its temperature will be relatively lower as depicted in Figure 12a.

The MVC feed temperature, *T_f_*, is the average of *T_fb_* and *T_fd_*; consequently, *T_f_* for S1 is higher than that for S2. As a result, D*T_f_* for S1 will be smaller than that for S2. This will have its implication on the required work and surface area, as will be discussed later in this section. Figure 12b illustrates how the water production grows for both cases with *T_b_*. The water production here is the sum of MVC and MD1 distillates for S1 and the sum of MVC, MD1, and MD2 distillates for S2. Note that the MVC distillate is the same for both cases; hence, the progression of water production is due to the contribution of MD1 and MD2. The evolution of *m_w_* with *T_b_* for both cases is expected as the MD performance enhances at higher inlet temperatures. Notably, *m_w_* for S2 evolves faster than that for S1, which is ascribed to the fact that *T_d_*, which comprises the energy source for MD2, progresses readily with *T_b_*. Nevertheless, the contribution of MD2 remains limited as the increment in production compared to S1 is 5% on average over the range of *T_b_*. The limited production of MD2 is attributed to two reasons. First, the feed flow rate to MD2 is less than that of MD1, i.e., *m_fd_* < *m_fb_*. It is known that the MD production increases with flow rate as higher circulation improves the hydrodynamics and heat transfer mechanism. Secondly, the temperature of the seawater fed to MD2 is reduced due to heat exchange in the distillate preheater. Figure 12c demonstrates the required specific compression work for both structures. Despite the increase in production rate, the specific work required by S2 is much larger than that of S1. On average, S2 demands 45% more compression energy. The reason is reflected in the temperature profile shown in Figure 12a. As S2 has a larger D*T_f_*, it will need more energy to maintain the MVC operation capacity of 1 kg/s distillate and brine of 70,000 ppm salinity. Nevertheless, the required specific heat transfer area is less for S2, as depicted in Figure 12d. On average, *sA_t_* for S2 is 43% less than that for S1. Although the S2 structure includes the surface area of the second MD unit, i.e., MD2, the latter is overshadowed by the evaporator transfer area, A_e_, and further normalized by the increased production rate. Thereby, the reduction in A_t_ for S2 is mainly due to a reduction in A_e_. Of course, the latter is another implication of the temperature profile shown in Figure 12a. Large D*T_f_* mandates a larger temperature drop across the MVC condenser tube and subsequently a smaller heat transfer area. The normalized area for S2 approaches that for S1 at very high *T_b_*; this is because at high brine temperature, a very large temperature drop at the condenser is needed to maintain the desired evaporation rate. Recall that the latent heat of vapor decreases with temperature.

## 5. Conclusions

In this work, membrane distillation is integrated with a mechanical vapor compression system for seawater desalination. The MD unit is integrated such that it utilizes the warm reject brine from the MVC to produce additional fresh water and cool down the reject brine. In this structure, the MD is operated using free input energy as it harnesses the thermal energy associated with the reject brine. Furthermore, it replaces the brine preheater to heat the seawater feed. It is found that the hybridization increases the water production rate by 5% at an operating temperature of 50 °C and by 10% at an operating temperature of 90 °C. Moreover, it reduces the required specific energy by an average of 32%. However, the required heat transfer area increased by an average of 60% due to the incorporation of the MD surface area. Altering the permeate-to-feed ratio in the MD unit caused little enhancement in the overall water production but no improvement in the required separation energy. Using a longer MD module with a 15 m^2^ surface area improved the overall water production by 3% and reduced the compression work by 10%. A modified structure that leverages the energy of the distillate stream is also examined. It is found that 5% growth in the water production and 43% reduction in the normalized surface area can be obtained but at the expense of 45% elevation in the compression work. Eventually, hybridizing MVC and MD improves the energy efficiency of each unit as well as the overall systems, leading to more sustainable design. Therefore, extending the present study to investigate other aspects such as exergy, cost, and environmental ones would give a more accurate and clear picture of the merits of MVC-MD hybridization. Moreover, optimizing the process parameters is important to achieve the optimum balance between energy consumption, capital cost and environmental impacts. As future works on the zero liquid discharge frame, further studies on optimization, exergy, and cost analysis on MD and MVC hybrids will be undertaken.

## Figures and Tables

**Table 1:** Operating condition of Ettouney et al. [40] process.

Parameter	Value
m_d_	1 kg/s
BPR	1 °C
T_cw_	30 °C
X_f_	42,000 ppm
X_b_	70,000 ppm
h	0.76
g	1.42
C_pv_	1.884 kJ/kg.K

**Table 2:** Membrane characteristics.

Parameter	Value
Effective surface area	10 m^2^
Membrane thickness	230 mm
Channel length	14 m
Channel height	0.7 m
Pore diameter	0.2 mm
Channel gap	0.2 mm
Porosity	0.8
Entry pressure	4.1 bar

## Data Availability

The original contributions presented in this study are included in the article. Further inquiries can be directed to the corresponding author.

## Nomenclature

*A_md_*	MD surface area area, m^2^
*A_b_*	Brine preheater surface area, m^2^
*A_d_*	Distillate preheater surface area, n^2^
*A_e_*	Evaporator surface area, m^2^
*A_t_*	Total surface area, m^2^
*BPE*	Boiling point elevation, C
*C_m_*	Permeability coefficient, kg/m^2^s Pa
$C_{m}^{k}$	Knudsen mass flux coefficient, kg/m^2^s Pa
$C_{m}^{d}$	Moléculaire diffusion mass flux coefficient, kg/m^2^s Pa
$C_{m}^{C}$	Transition mass flux coefficient, kg/m^2^s Pa
*C*_p_	Heat capacity, J/kg K
*de*	collision diameter of the water vapor and air, m^2^
*h*_v_	Latent heat of vaporization for MD, J/kg
*h*_f_, *h*_p_, *h*_m_	Feed, permeate, and membrane heat transfer coefficient, W/m^2^ K
$H_{v}$	Enthalpy of vapor at $T_{v}$, kJ/kg
$H_{s}$	Enthalpy of superheated steam at $T_{s}$, kJ/kg
*J_w_*	Mass flux, kg/m^2^ h
*k_B_*	Boltzmann constant
*k*_m_	Membrane conductivity, W/m K
*k_s_*	Solid phase thermal conductivity, W/m.K
*k_g_*	Gas phase thermal conductivity, W/m.K
*k_n_*	Knudsen number
*l*	Channel height, m
*LMDT*	Logarithmic mean temperature difference
$m_{d}$	Distillate mass rate for MVC, kg/s
$m_{b}$	MVC brine flow rate, kg/s
$m_{f}$	Seawater intake rate, kg/s
${m_{f}}_{b}$	Seawater stream fed to brine preheater/MD unit, kg/s
${m_{f}}_{d}$	Seawater stream fed to distillate preheater, kg/s
$m_{w}$	distillate mass rate for MD, kg/s
${m_{d}}_{t}$	Total distillate mass rate, kg/s
M_w_	Molecular weight
*Nu*	Nusselt Number
P_1_, P_2_	Vapor pressure at feed and permeate membrane surfaces, Pa
P	Average membrane interface pressure, Pa
P_a_	Entrapped air pressure, Pa
PD	Membrane pressure multiplied by diffusivity, Pa.m^2^/s
Pr	Prandtl number
$P_{v}$	Vapor pressure at $T_{v}$, Pa
$P_{d}$	Vapor pressure at $T_{d}$, Pa
$Q_{e},\;Q_{c}$	Evaporator and condenser heat load, kJ/s
$Q_{h}$	Preheater heat load, kJ/s
*r*	Membrane pore size, m
R	Ideal gas constant, also flow rate ratio
*Re*	Reynold Number
$sA_{t}$	Specific total surface area, m^2^/(kg/s)
sW	Specific compressor work, kWh⋅m^−3^
*T*_h_, *T*_c_	Feed (hot) and permeate (cold) temperature, K
*T_hb_*, *T_cb_*	Feed (hot) and permeate (cold) bulk temperature, K
*T*_hm_, *T*_cm_	Feed and permeate membrane temperature, K
${T_{f}}_{b}$	Seawater temperature exiting brine preheater, C
${T_{f}}_{d}$	Seawater temperature exiting brine preheater, C
T	The average temperature at the membrane interface, K
$T_{f}$	MVC feed temperature, C
$T_{d}$	Distillate temperature, C
$T_{s}$	Temperature of the superheated steam, C
$T_{v}$	Vapor temperature, C
$T_{o}$	Temperature exiting preheaters, C
$T_{cw}$	Seawater intake temperature, C
*U*	Overall heat transfer coefficient, W/m^2^K
$V_{v}$	Vapor specific volume, m^3^/kg
$X_{f},X_{b}$	Feed and brine salinity, ppm
W	Actual Compressor work, kW
$W_{a}$	Adiabatic compressor work, kW
*Greek letters*	
h	Compressor efficiency
$\eta_{h}$	Heat exchanger efficiency
τ	tortuosity
ρ	Water density, kg/m^3^
δ	Membrane thickness
ε	porosity
g	Heat capacity ratio
λ	Mean free path, m
l_d_	Latent heat at $T_{d}$, kJ/kg
l_v_	Latent heat at $T_{v}$, kJ/kg

## Appendix A

This section presents the solution procedure of the MD model. The following formulation is the summary of our previous work with MD modeling and analysis [43,44,45]. To start the procedure, the flow rate, temperature, and salinity of the feed and permeate stream should be specified ($m_{b},\;m_{fb},\;T_{b},\;T_{cw},\;X_{b},\;X_{f}$). For simplicity, the inlet temperatures will be taken as the bulk temperatures, i.e., ${T_{h}}_{b}=T_{b},\;{T_{c}}_{b}=T_{cw}$

1. Given the bulk temperature at both sides of the MD membrane (${T_{h}}_{b},\;{T_{c}}_{b}$) the local heat transfer coefficients ($h_{f},\;h_{p}$) are calculated from the Nusselt number as follows [37]:

(A1) $$Nu=0.298{Re}^{0.646}{Pr}^{0.316}$$

where Re is Reynolds number and Pr is Prandtl number.

2. Set ${T_{h}^{0}}_{m}={T_{h}}_{b}\;and\;{T_{c}^{0}}_{m}={T_{c}}_{b}$
3. Calculate the vapor pressure at the membrane interface using [36]:

(A2)
$$P_{1}=exp(23.238-\frac{3841}{T_{hm}-45})(1-X_{f}\times 10^{-6})$$

(A3)
$$P_{2}=exp(23.238-\frac{3841}{T_{cm}-45})\;(1-X_{b}\times 10^{-6})$$

4. Knowing the membrane characteristics and the average membrane temperature, i.e., $T=\frac{{T_{h}}_{m}+{T_{c}}_{m}}{2}$, the membrane coefficient *C*_m_ can be estimated utilizing the correlation in [50] according to the designated mechanism:

- Knudsen flow mechanism, *k*_n_ > 1:

(A4)
$$C_{m\;}^{k}=\frac{2\varepsilon r}{3\tau \delta }{\left(\frac{8M_{w}}{\pi RT}\right)}^{1/2}$$

- Molecular diffusion mechanism, *k*_n_<0.01:

(A5)
$$C_{m}^{D}=\frac{\varepsilon }{\tau \delta }\frac{PD}{P_{a}}\frac{M_{w}}{RT}$$

- Knudsen-molecular diffusion transition mechanism, 0.01 < *k*_n_ < 1:

(A6) $$C_{m\;}^{C}={\left[{\frac{3}{2}\frac{\tau \delta }{\varepsilon r}\left(\frac{\pi RT}{8M_{w}}\right)}^{1/2}+\frac{\tau \delta }{\varepsilon }\frac{P_{a}}{PD}\frac{RT}{M_{w}}\right]}^{-1}$$

where Knudsen number is defined as $k_{n}=\frac{\lambda }{d}$ and λ is the mean free path of water molecules, expressed as [37]:

(A7)
$$\lambda =\frac{k_{B}T}{\sqrt{2}\pi P{d_{e}}^{2}}$$

P is the average pressure at the membrane interface, respectively, *k*_B_ = 1.380622 × 10^−23^ and *d*_e_ = 9.29 × 10^−20^

5. Calculate the latent heat of vaporization at the average membrane temperature using [49]:

(A8)
$$h_{v}\left(T\right)=1850.7+2.8273T-1.6\times 10^{-3}T^{2}$$

6. Calculate the mass flux using:

(A9)
$$j_{w}=C_{m}\left(P_{1}-P_{2}\right)$$

7. Compute the overall heat transfer coefficient using [11]:

(A10)
$$U={\left[\frac{1}{h_{f}}+\frac{1}{h_{m}+\frac{J_{w}h_{v}}{T_{h_{m}}-T_{c_{m}}}}+\frac{1}{h_{p}}\right]}^{-1}$$

The membrane heat transfer coefficient (*h*_m_) represents the heat resistance due to conduction and can be estimated using [60]:

(A11) $$h_{m}=\frac{{\;k}_{m}}{\delta }=\frac{\left(1-\varepsilon \right)k_{s}+\varepsilon k_{g}}{\delta }$$

8. At equilibrium, the heat convection from the hot side to the membrane interface and heat convection from the membrane interface to the cold side are equal. Hence the following conditions hold [50]:

(A12)
$$U\left(T_{h_{b}}-T_{c_{b}}\right)=h_{f}\left(T_{h_{b}}-T_{h_{m}}\right)=j_{w}h_{v}+h_{m}(T_{h_{m}}-T_{c_{m}})$$

(A13)
$$U\left(T_{h_{b}}-T_{c_{b}}\right)=h_{p}\left(T_{c_{m}}-T_{c_{b}}\right)=j_{w}h_{v}+h_{m}(T_{h_{m}}-T_{c_{m}})$$

These constraints can be used to compute new values for $T_{h_{m}}\;and\;T_{c_{m}}$

9. If $T_{h_{m}}=T_{h_{m}}^{0}\;and\;T_{c_{m}}=T_{c_{m}}^{0}$ then stop the iteration, otherwise set $T_{h_{m}}^{0}=T_{h_{m}}\;and\;T_{c_{m}}^{0}=T_{c_{m}}$ and go back to step 3.

When the algorithm converges, the water production is computed as follows:

(A14) $$m_{w}=j_{w}\times A_{md}$$

The outlet temperatures are calculated from the following heat balance around the MD module for the hot side and cold side as follows:

(A15) $$m_{b}Cp_{b}\left(T_{b}-T_{o}\right)=U\times A_{md}\times (T_{h_{b}}-T_{c_{b}})$$

(A16) $$m_{fb}Cp_{fb}\left(T_{fb}-T_{cw}\right)=U\times A_{md}\times (T_{h_{b}}-T_{c_{b}})$$
